# Muzzling the School Doctor

**Published:** 1912-01-27

**Authors:** 


### MUZZLING THE SCHOOL DOCTOR.

We hear with considerable astonishment that the fiat has gone forth from the Board of Education that school medical officers, engaged in the inspection of school children and drawing salaries from local school authorities, are henceforth not to publish any details concerning their work. In other words, a stop is to be put to the illuminative and educative reports which some school doctors, whose scientific spirit has not been wholly swallowed up by the drudgery of routine work, have from time to time published in the medical and lay journals. We confess that we entirely fail to see the reason for such a prohibition. Nothing but good can result from the free discussion of faults met with and defects noted. What is wanted at present is the honest compilation of statistics, the equally unprejudiced publication of experiences, and the impartial reporting of conditions noted. It will be a great pity if these reports are to be treated as confidential and submitted only to the medical officer of the Board of Education, for him to use, *in précis*, in his annual *résumé* of results. To begin with, that annual *résumé* is in itself usually so stereotyped and so overloaded with extraneous material that it escapes the full review and criticism which are given to shorter and more individual reports. Secondly, many men are interested in special features of school medical work: they are specially qualified to deal with these aspects in a manner that the chief medical officer—we say it with all respect—is not. It stands to reason that most of us will pay more attention to an article dealing with, say, the cardiac condition of school children written by a specialist, who has the right to claim a hearing when he speaks on this subject by reason of the fact that he has specially identified himself with this matter, than we would to a similar article appearing as an appendix to the annual report of the medical officer of the Board of Education.

That being the case, we feel that it is imperative for medical officers of schools energetically to protest against this contemplated interference with their privileges of free speech and writing by the Board. It is true every school doctor who is on the staff of a hospital will easily find a way out of the difficulty by disregarding the prohibition and dealing with his child patients as if they were his hospital patients. But his colleagues who are not so favoured will not have a similar loophole of escape, and they will scarcely be blamed if they shrink from infringing the command of the authorities and suppress their notes. In that case the profession stands to lose a good deal by the suppression of that personal report of experience and work which is so valuable. It is to be hoped therefore that some authoritative and representative body will take up the matter and lead the protest. The School Medical Officers' Association is the best body to take the lead in this matter, and we look to it to safeguard the rights of its members and of the profession generally by energetically protesting against the new commandment—a commandment which is as much against the interests of the general public as it is against the interests of the profession.

It is perhaps lucky that this prohibition has been issued at a time when the whole medical profession is astir in defence of its rights in another direction. There is the less chance of any school doctor taking this new attack "lying down." There should be absolutely no hesitancy on the part of medical men in protesting against this muzzling, for the present attempt is one made not merely on their professional rights, but on the general rights of free speech that are usually claimed as irrefragibly guaranteed to every citizen. To this second attack on its prerogatives the profession should show an undivided front, for if that is maintained the Board of Education will be compelled to retreat from this position. Now is the time for the School Medical Officers' Association to prove its value by organising the protest. By so doing it will deserve, and gain, the whole-hearted respect of every man in the profession.

